# Towards a network control theory of electroconvulsive therapy response

**DOI:** 10.1093/pnasnexus/pgad032

**Published:** 2023-02-01

**Authors:** Tim Hahn, Hamidreza Jamalabadi, Erfan Nozari, Nils R Winter, Jan Ernsting, Marius Gruber, Marco J Mauritz, Pascal Grumbach, Lukas Fisch, Ramona Leenings, Kelvin Sarink, Julian Blanke, Leon Kleine Vennekate, Daniel Emden, Nils Opel, Dominik Grotegerd, Verena Enneking, Susanne Meinert, Tiana Borgers, Melissa Klug, Elisabeth J Leehr, Katharina Dohm, Walter Heindel, Joachim Gross, Udo Dannlowski, Ronny Redlich, Jonathan Repple

**Affiliations:** Institute for Translational Psychiatry, University of Münster, 48149 Münster, Germany; Department of Psychiatry and Psychotherapy, University of Tübingen, 72076 Tübingen, Germany; Department of Mechanical Engineering, University of California, 92521 Riverside, USA; Institute for Translational Psychiatry, University of Münster, 48149 Münster, Germany; Institute for Translational Psychiatry, University of Münster, 48149 Münster, Germany; Faculty of Mathematics and Computer Science, University of Münster, 48149 Münster, Germany; Institute for Translational Psychiatry, University of Münster, 48149 Münster, Germany; Institute for Translational Psychiatry, University of Münster, 48149 Münster, Germany; Institute for Translational Psychiatry, University of Münster, 48149 Münster, Germany; Institute for Translational Psychiatry, University of Münster, 48149 Münster, Germany; Institute for Translational Psychiatry, University of Münster, 48149 Münster, Germany; Faculty of Mathematics and Computer Science, University of Münster, 48149 Münster, Germany; Institute for Translational Psychiatry, University of Münster, 48149 Münster, Germany; Institute for Translational Psychiatry, University of Münster, 48149 Münster, Germany; Institute for Translational Psychiatry, University of Münster, 48149 Münster, Germany; Institute for Translational Psychiatry, University of Münster, 48149 Münster, Germany; Institute for Translational Psychiatry, University of Münster, 48149 Münster, Germany; Institute for Translational Psychiatry, University of Münster, 48149 Münster, Germany; Institute for Translational Psychiatry, University of Münster, 48149 Münster, Germany; Institute for Translational Psychiatry, University of Münster, 48149 Münster, Germany; Institute for Translational Neuroscience, University of Münster, 48149 Münster, Germany; Institute for Translational Psychiatry, University of Münster, 48149 Münster, Germany; Institute for Translational Psychiatry, University of Münster, 48149 Münster, Germany; Institute for Translational Psychiatry, University of Münster, 48149 Münster, Germany; Institute for Translational Psychiatry, University of Münster, 48149 Münster, Germany; Institute of Clinical Radiology, University of Münster, 48149 Münster, Germany; Institute for Biomagnetism and Biosignalanalysis, University Hospital Münster, 48149 Münster, Germany; Institute for Translational Psychiatry, University of Münster, 48149 Münster, Germany; Institute for Translational Psychiatry, University of Münster, 48149 Münster, Germany; Department of Psychology, University of Halle, 06099 Halle (Saale), Germany; Institute for Translational Psychiatry, University of Münster, 48149 Münster, Germany

**Keywords:** network control theory, diffusion tensor imaging, electroconvulsive therapy, major depressive disorder, postictal suppression index

## Abstract

Electroconvulsive Therapy (ECT) is arguably the most effective intervention for treatment-resistant depression. While large interindividual variability exists, a theory capable of explaining individual response to ECT remains elusive. To address this, we posit a quantitative, mechanistic framework of ECT response based on Network Control Theory (NCT). Then, we empirically test our approach and employ it to predict ECT treatment response. To this end, we derive a formal association between Postictal Suppression Index (PSI)—an ECT seizure quality index—and whole-brain modal and average controllability, NCT metrics based on white-matter brain network architecture, respectively. Exploiting the known association of ECT response and PSI, we then hypothesized an association between our controllability metrics and ECT response mediated by PSI. We formally tested this conjecture in N = 50 depressive patients undergoing ECT. We show that whole-brain controllability metrics based on pre-ECT structural connectome data predict ECT response in accordance with our hypotheses. In addition, we show the expected mediation effects via PSI. Importantly, our theoretically motivated metrics are at least on par with extensive machine learning models based on pre-ECT connectome data. In summary, we derived and tested a control-theoretic framework capable of predicting ECT response based on individual brain network architecture. It makes testable, quantitative predictions regarding individual therapeutic response, which are corroborated by strong empirical evidence. Our work might constitute a starting point for a comprehensive, quantitative theory of personalized ECT interventions rooted in control theory.

**Significance Statement**

Electroconvulsive Therapy (ECT) is the most effective treatment for treatment-resistant major depressive disorder (MDD), but not all patients respond to this treatment. Here, we employ control theory—an approach originally developed to investigate the control of dynamical systems in engineered processes and machines—and apply it to patients’ individual structural brain networks. We show that the effective suppression of an ECT-induced seizure depends on brain network control properties and that differences in these control metrics explain differential clinical responses in MDD patients. Therefore, brain images before the start of the treatment could aid in the prediction of treatment response and help explain the underlying mechanisms of this effective but poorly understood treatment.

## Introduction

Electroconvulsive therapy (ECT) is the most effective treatment for severe and therapy-resistant depression (1–3). As therapeutic response varies widely, however, numerous studies have sought to explain individual ECT response from indexes, rating scales, or symptom clusters prior to intervention, among others (4–7). More recently, although limited to small patient samples, more machine learning models based on neuroimaging data have rekindled hopes for a theranostic biomarker (8–11). These efforts, although useful to identify potential predictive factors of ECT response, however, cannot directly add to a theoretical framework capable of explaining ECT response because of their purely data-driven and predictive nature.

Generally, during ECT an electric charge is applied to the brain to induce a generalized tonic–clonic seizure characterized by high amplitudes and polyspike waves as well as high amplitudes with slow waves in the electroencephalogram (EEG). This is followed by the termination phase during which postictal suppression—i.e. a period of lower spectral amplitude and a general flattening of the EEG—occurs (12). Empirically, postictal suppression has repeatedly been linked to treatment response: A plethora of studies for more than three decades has found a positive association between stronger postictal suppression and better response to ECT (12–23). Recognizing this, ECT devices today provide a measure of postictal suppression which is routinely used in clinical practice to assess treatment quality and estimate future therapeutic response. This postictal suppression index (PSI) is quantified by the ratio of EEG signal power during the termination phase and the tonic–clonic seizure and subtracting it from 1 (see Eq. 1 in Methods).

While empirically well-founded and routinely used in the clinic, a theoretical framework explaining why ECT patients vary with regard to seizure quality indices such as PSI is, however, missing. Consequently, PSI is of limited theranostic utility as it can only be measured during ECT—i.e. after the beginning of the intervention—and thus cannot serve as a predictive marker in therapeutic planning. In contrast, identifying the interindividual differences underlying the observed variance in PSI would allow us to derive a predictive marker for ECT response suitable for therapeutic planning.

To enable the theoretically driven construction of a theranostic biomarker, we conceptualize ECT as an attempt to drive the brain into a specific state (i.e. seizure) by influencing large-scale, dynamic network state transitions in the brain. Building on Control Theory as the study and practice of controlling dynamical systems (24), we can then view the electric charge applied during ECT as a control input designed to guide the system towards a specific state—i.e. a seizure—characterized by the high amplitude EEG oscillations typical for tonic–clonic seizures.

Next, we aim to relate this control-theoretic perspective to network neuroscience: Specifically, recent progress in Network Control Theory (NCT) has enabled the quantification of the influence a brain region (or “node” from a network perspective) has on the dynamic transitions between brain states (i.e. the multivariate pattern of neural activity across the whole brain) (25, 26). In other words, from a theoretical perspective, a brain region with higher controllability, if stimulated, ought to have a greater effect on brain dynamics than a region with lower controllability. In this context, controllability of a brain region is an important metric that links stimulation (e.g. ECT), the brain state after stimulation (i.e. a seizure), and the brain's structural connectivity properties (27, 28). Importantly, controllability is an abstract concept that can be operationalized in different ways: Most commonly, controllability is captured by two key metrics: On the one hand, *modal controllability* represents the ability of brain regions to especially affect the trajectory of fast decaying neural dynamics i.e. those brain activities that last only a very short amount of time (29). On the other hand, *average controllability* measures the ability of a system to generally spread and amplify intervention effects (i.e. the electric charge used during ECT) and is thus indicative of a brain region's average ability to induce a large number of different neural patterns (for formal definitions of *average* and *modal controllability* measures see Methods section). Pertaining to brain dynamics, it has been shown that average controllability is highest in those brain regions that have a higher number of structural connections (i.e. structural degree) and higher gray matter volume while modal controllability is highest in those regions with a lower number of structural connections and lower gray matter volume (27). Fueled by evidence that (1) the human brain is in principle controllable (27) (i.e. external stimulation of the brain can indeed change brain dynamics in a meaningful and arbitrarily complex way), (2) control-theoretic constructs are directly linked to biological mechanisms in the brain (30), and (3) controllability is associated with cognition (28, 31), numerous studies have empirically investigated the two metrics in mental disorders (32–34). Focusing specifically on Major Depressive Disorder (MDD), Hahn et al. recently showed in a large sample of patients that whole-brain average and modal controllability (i.e. mean average and modal controllability across the brain) is related to genetic, individual, and familial risk in MDD patients (35).

In the current study, we formally derive from control theory that whole-brain modal and average controllability are mathematically related to amplitude of global brain response to control input (i.e. output signal power after a control input): As whole-brain average controllability is defined as the weighted sum of the so-called impulse response of each brain area, it is directly related to the cumulative power of the output response (i.e. the EEG signal power during the seizure). Likewise, whole-brain modal controllability is linked to the observed brain dynamics after inducing energy into the system. For a formal derivation of these properties, see online Supplementary material Methods section.

In other words, if we conceptualize the electric charge applied during ECT as a control input designed to induce a seizure, it follows that whole-brain average and modal controllability derived from the structural connectome are expected to be related to EEG signal power after control input (i.e. during the induced seizure). Specifically, higher whole-brain average and lower modal controllability ought to be related to higher output signal power during the tonic–clonic seizure (see *Network Control Analysis* in the Methods section for a more stringent argument). Importantly, as PSI is proportional to the ratio of signal power during the termination phase and the tonic–clonic seizure (see Eq. 1 in Methods), higher signal power during the tonic–clonic seizure phase should result in higher PSI.

In summary, we posit that lower whole-brain modal controllability and higher whole-brain average controllability (both mathematically associated with higher output signal power during the tonic–clonic seizure as formally derived using the principles of Control Theory) should lead to higher PSI values. As the positive relationship between PSI and therapeutic response is well documented, it follows that lower whole-brain modal controllability and higher whole-brain average controllability, respectively, should result in higher PSI and thus improved ECT response. Here, we empirically test this conjecture by assessing the relation between empirically observed PSI during ECT and whole-brain modal and average controllability derived from pre-ECT structural connectome data in N = 50 naturalistically recruited participants diagnosed with current MDD and treated with ECT (see Methods section for details). Next, we replicate the association between PSI and ECT response known from the literature. Then, we test whether pre-ECT whole-brain modal and average controllability predict ECT response and assess whether these effects are indeed mediated by PSI during ECT. Finally, we compare the predictive power of whole-brain modal and average controllability regarding therapeutic response to the performance of an extensive array of machine learning models based on structural connectome data.

## Results

To empirically test our hypotheses derived via the application of control theory to ECT, we conducted four analyses: First, we assessed the effect of the empirically observed whole-brain modal controllability ($\bar{MC}$) and whole-brain average controllability ($\bar{AC}$), respectively, on PSI during ECT. As hypothesized, $\bar{MC}\;$ is negatively associated with PSI (F(1,39) = 3.28, *P* = 0.039, partial η^2^ (ηp^2^) = 0.077). Likewise, $\bar{AC}$ shows a trend-wise positive association with PSI (F(1,39) = 1.72, *P* = 0.098, ηp^2^ = 0.042).

Second, we replicated the association between higher PSI and better ECT response known from the literature (F(1,39) = 7.28, *P* = 0.005, ηp^2^ = 0.157).

Third, we tested whether pre-ECT $\bar{MC}$ and $\bar{AC}$ predicted ECT response. In line with our hypotheses, we reveal that lower $\bar{MC}$ is indeed associated with stronger ECT response (F(1,44) = 8.15, *P* = 0.004, ηp^2^ = 0.156). Furthermore, as expected, $\bar{AC}$ is positively related to ECT response (F(1,44) = 3.62, *P* = 0.032, ηp^2^ = 0.076).

**Fig. 1.**
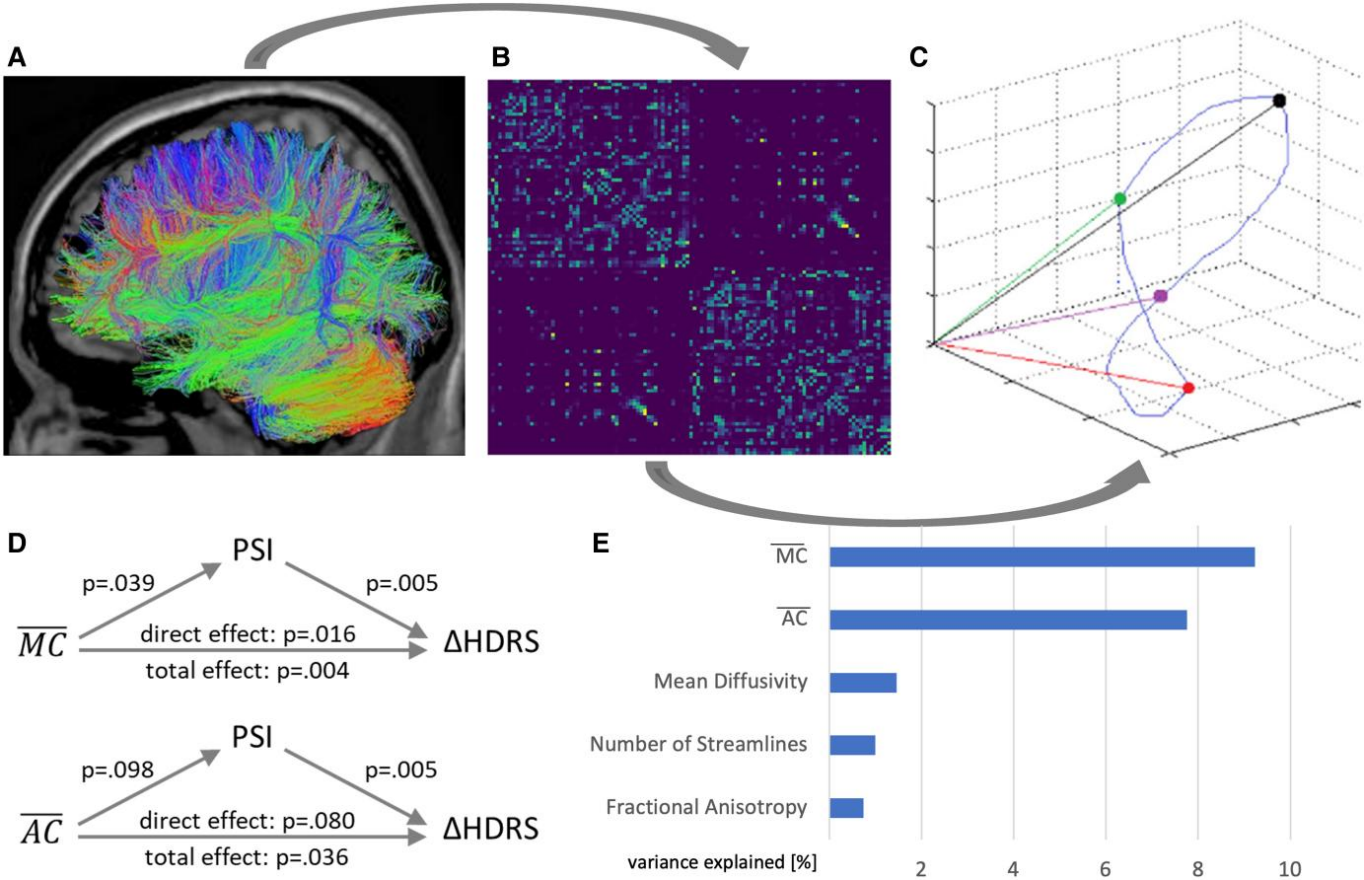
From Diffusion Tensor Imaging (DTI) data (A), we derived the structural connectivity matrix for each patient (B) and quantified modal and average controllability—i.e. the influence a brain region has on the dynamic transitions between brain states underlying cognition and behavior (illustrated in C). We then show that mean modal ($\bar{MC}$) and average controllability ($\bar{AC}$), respectively, are associated with therapeutic response to ECT treatment and that this effect is mediated by Postictal Suppression Index (PSI; D). $\bar{MC}$ and $\bar{AC}$ also predict therapeutic response in patients before ECT treatment as good or better than an extensive array of multivariate machine learning models based on Fractional Anisotropy, Mean Diffusivity, and Number of Streamlines derived from DTI (E).

Fourth, we tested if the effects of $\bar{MC}$ and $\bar{AC}$, respectively, on ECT response are indeed mediated by PSI. To this end, we employed a mediation analysis with $\bar{MC}$ and $\bar{AC}$, respectively, as the predictor, PSI as the mediator, and ECT response as the outcome (Fig. 1D). We indeed observed a significant mediation (i.e. indirect) effect for $\bar{MC}$ (ab = 184.80; *P* = 0.010) as well as for $\bar{AC}$ (ab = −47.94; *P* = 0.017). However, a significant direct effect (c′)—i.e. an association between $\bar{MC}$ with ECT response after controlling for PSI—remains (c′ = 706.26; *P* = 0.016); indicating a relation to ECT response above and beyond the PSI-mediated effect. In contrast, the effect of $\bar{AC}$ on ECT response is fully mediated by PSI, i.e. it no longer reaches significance when controlling for PSI (c′ = −140.40; *P* = 0.080).

Finally, we descriptively compared the predictive power of $\bar{MC}$ and $\bar{AC}$ regarding ECT response to the performance of an extensive array of machine learning models based on structural connectome data. Specifically, we tested 35 combinations of data transformations (including Principal Component Analysis and univariate feature selection) and multivariate machine learning algorithms (including linear and non-linear Support Vector Machines, Random Forests, and linear Regression Models) in a nested cross-validation approach using Fractional Anisotropy, Mean Diffusivity, and Number of Streamlines derived from each patient's structural connectome, respectively (see Methods for details). We showed that $\bar{MC}$ (r^2^ = 9.24%) as well as $\bar{AC}$ (r^2^ = 7.76%), respectively, explain nominally more variance in treatment response than the best machine learning model for each modality (Fractional Anisotropy r^2^ = 0.74%; Mean Diffusivity r^2^ = 1.46%; Number of Streamlines r^2^ = 0.99%; Fig. 1E). Also, controllability metrics outperformed a model combining age, gender, and symptom severity at baseline (r^2^ = 4.37%).

## Discussion

In this work, we drew upon NCT to derive a mechanistic framework capable of predicting ECT response based on individual, pre-ECT white-matter architecture. This approach has two main strengths: First, unlike previous efforts in psychiatry, it allows us to derive testable predictions regarding individual therapeutic response from a well-established, quantitative theory, which can further inform mechanistic models of ECT response. Second, these theoretically derived predictions—which, unlike PSI, can be obtained before ECT and can thus be used for treatment planning—are at least on par with state-of-the-art multivariate machine learning approaches, hence opening a new paradigm to study ECT response. On a mathematical level, our approach essentially builds upon the notion of controllability which relates the geometrical properties of brain structural connectome to the functional role each brain region can play in steering the brain dynamics. Conceptualizing ECT as an intervention to a networked system (i.e. the brain), we formulate a mathematical framework that relates structural brain networks to the PSI and thus clinical outcomes of ECT on depressive symptomatology.

Specifically, we first derive that lower whole-brain modal controllability and higher whole-brain average controllability are mathematically associated with higher output signal power during the tonic–clonic seizure if we conceptualize the electric charge applied during ECT as the control input to a noise-free, time-invariant linear system commonly used in NCT and the seizure as its output. Based on the calculation of PSI as routinely done in the clinical context, we can then directly deduce that—all other things being equal—PSI should increase for patients with lower $\bar{MC}$ and higher $\bar{AC}$, respectively. Drawing on previous evidence showing a positive association between PSI and ECT response, we then hypothesize higher ECT response for patients with lower $\bar{MC}$ and higher $\bar{AC}$, respectively. All three effects predicted by our approach for $\bar{MC}$—i.e. the associations between 1. $\bar{MC}$ and PSI, 2. PSI and ECT response, and 3. $\bar{MC}$ and ECT response—as well as the mediation effect of PSI, were empirically found in the theoretically expected directions. For $\bar{AC}$, results were also in the expected direction, but effects were smaller (ηp^2^ of 0.068 and 0.076) and in case of the association of $\bar{AC}$ and PSI only marginally significant. These findings support the notion that ECT response—at least in part—can be understood analogous to physical dynamical systems within the formal framework of NCT viewing the electric charge applied during ECT as the control input and the ECT-induced seizure as the control output.

Importantly, a comparison with state-of-the-art machine learning approaches showed that a simple linear predictive model using only the single $\bar{AC}$ or $\bar{MC}$ value, respectively, for each patient is at least as good a predictor of therapeutic response as an extensive array of 35 machine learning pipelines based on the multivariate patterns of Fractional Anisotropy, Mean Diffusivity, and Number of Streamlines derived from each patient's structural connectome. In the same vein, we know from epidemiological data that disease severity before treatment, age, and sex are predictors of ECT response. Interestingly, if we use this information in a multivariate model using our dataset, the model can only explain around 4% of variance. In contrast, the controllability metrics can explain 8–9% of the variance, which demonstrates the substantial role that these metrics could play in future development of formal ECT response prediction models in clinical practice. Generally, this shows that our theoretically derived metrics are comparable or better predictors of treatment response than an extensive array of state-of-the-art multivariate machine learning approaches or demographic information known to be associated with ECT response—thus encouraging further research for theranostic markers in this direction.

More generally, connecting NCT and ECT opens the door towards a quantitative understanding of ECT response. Providing a quantitative answer to the question of what changes in the brain after a specified stimulation event is not only crucial for ECT response prediction, but also—with NCT—provides a rich theoretical framework with which we can hope to optimize ECT application itself. For example, our results are conceptually related to successful attempts to predict stimulation outcome in the context of electrical brain stimulation (36). While demonstrating that variation in response to treatment can be explained by controllability differences, our approach, however, relies exclusively on whole-brain controllability metrics—not local controllability of certain brain regions. While we think this is particularly reasonable in the context of ECT, which induces a generalized seizure across the entire brain, future studies ought to extend the idea to include localized control. This is particularly interesting as *NCT* provides a straightforward way to calculate which brain regions should be targeted in which order to efficiently reach a specific state (be it a seizure or other states) employing more localized interventions such as Transcranial Magnetic Stimulation. Whether targeting regions identified by this so-called control-node analysis (37) leads to stronger response can be directly tested in future intervention studies. Importantly, this view also entails that therapeutic interventions should be custom-tailored to the patient's individual structural connectome topology which might thus serve as a promising approach to patient stratification.

In addition to the prediction of ECT response, a control-theoretic framework might help to understand several phenomena associated with ECT. For example, inducing seizures becomes increasingly difficult with age which might be explained by the fact that synchronizability—i.e. the ability of the constituents of a dynamical system to show coherent activity—develops in a way as to favor seizure suppression over the lifespan (38). Also, the same study shows that strong modal controllers were disproportionately located in cognitive control systems, including both the frontoparietal and cingulo-opercular systems. This might entail that the efficacy of ECT and its adverse effects regarding cognitive deficits maybe linked at the level of white-matter network architecture.

Several limitations should be noted. First, calculation of average and modal controllability relies on the simplified noise-free linear discrete-time and time-invariant network model employed in virtually all work on brain NCT (27, 39, 40). Given the brain's clearly non-linear dynamics, this is justified as (1) non-linear behavior may be accurately approximated by linear behavior (41, 42) and (2) the controllability of linear and non-linear systems is related such that a controllable linearized system is locally controllable in the non-linear case (see also (27) for details). If a more complex non-linear model could improve the results presented here should be tested in future studies.

Second, we formalized our model by conceptualizing the seizure as the brain's response to ECT. This is motivated by the observations that it is indeed the seizure that seems to be central to the clinical efficacy of ECT. For example, a multitude of evidence is accumulating from the newly developed magnetic seizure therapy (43), low-dose electrical shock therapy (44), and sham therapies that simulate (45). PSI is however, by definition, also related to the ictal EEG. How and if the inclusion of this period can improve the current methodology should be studied in future research.

Third, our estimation of controllability is based upon binary networks built from streamline counts of DTI tractography. This introduces two possibilities to improve the current study. On the one hand, DTI itself is limited in its ability to accurately quantify the structural connectome (for an introduction, see (46)). Currently, several novel approaches to controllability quantification are being explored including estimation from gray matter (47) and resting-state functional dynamics (39). The inclusion of these methodologies to infer the brain networks embodying brain dynamics could in principle improve our model. For example, the connectivity networks could be derived based on a number of different methodologies including weighted graphs based on the number streamlines. Related, although a fundamental concept in engineering studies, controllability remains an abstract concept in studying brain dynamics. Recent studies have shown that regional controllability is closely related to the and potentially operationalized by regional metabolic dynamics (30), gray matter volume (47), and the geometrical properties of structural connectome (27). Empirically comparing and theoretically reconciling results from these methods will be crucial for robust parameter estimation in NCT studies of the brain. In addition, longitudinal data from DTI, gray matter, and resting-state functional dynamics available from e.g. the Marburg–Münster Affective Disorders Cohort Study (MACS; (48)) will enable us to assess the (differential) reliability of these approaches. In combination with functional Magnetic Resonance Imaging, this also provides an opportunity to further characterize the relationship between network control and individual task-related activation (49).

In summary, we derived and tested a control-theoretic framework capable of predicting ECT response based on individual brain network architecture. It makes testable, quantitative predictions regarding individual therapeutic response, which are corroborated by strong empirical evidence. Our work might constitute a starting point for a comprehensive, quantitative theory of personalized ECT interventions rooted in control theory.

## Methods

### Sample

Fifty-three participants diagnosed with current MDD and treated with ECT participated in the present study. Patients were recruited naturalistically with treatment being assigned based on clinical decisions independent from study participation. All subjects were diagnosed with the Structured Clinical Interview for DSM-IV-TR (50) to confirm the psychiatric diagnosis. For medication details and study inclusion and exclusion criteria, see online supplementary material. In the process of image quality control (see Methods section below), three subjects had to be excluded. Therefore, the final sample comprised of 50 subjects (29 female, 21 male; mean age = 45.1 years, SD = 10.8). Note that results do not substantially change if these three subjects are not excluded. This study was approved by the ethics committee of the Medical Faculty of Muenster University and all subjects gave written informed consent prior to participation. They received financial compensation after study completion.

For MRI data acquisition and preprocessing, see online Supplementary material Methods.

### Electroconvulsive therapy

Brief-pulse ECT was conducted three times a week using an integrated instrument (Thymatron IV; Somatics Inc; number of sessions: *M* = 13.0, *SD* = 4.34, range = 5–25). Energy dosage elevation was considered between ECT sessions if the primarily induced seizure activity lasted less than 25 s. For more details on ECT procedure and parameters see online Supplementary material.

Recognizing the empirical link between PSI and ECT response, many ECT devices today provide a measure of postictal suppression which is routinely used in clinical practice to assess seizure quality and estimate future therapeutic response. During ECT, the ictal outcome of PSI was measured and summarized by the Thymatron machines using five electrodes. These were placed on the right and left forehead, on the right and left mastoid, and on the patient's nasion (ground electrode). The PSI is quantified by the ratio of signal power during the termination phase and power during the tonic–clonic seizure based on the EEG time-series and subtracting it from 1:

(1) $$PSI=\left(1-\frac{Power_{termination\;phase}}{Power_{tonic-clonic\;seizure}}\right)\propto Power_{tonic-clonic\;seizure}$$

It follows that higher EEG signal power during the tonic–clonic seizure should result in higher PSI values.

We used Thymatron system IV's default settings to compute PSI. Specifically, signal power during the termination phase and the seizure, respectively, is computed as the mean power of three 1.28 s long intervals with an EEG sampling rate of 200 Hz. To minimize artifacts, 3.84 s around the tonic–clonic seizure endpoint are disregarded (F. Berninger, Somatics Inc. representative, personal e-mail communication with JR, 2017 December 15).

### Choice of primary measure

In this work, we used ECT response as the primary outcome measure. MDD symptoms at both time points were measured using the Hamilton Depression Rating Scale (HDRS) (51). ECT response was defined as the difference in MDD symptoms before and after ECT. Specifically, we subtracted pre-ECT scores from post-ECT scores so that improvement is indicated by more negative values. Despite concerns regarding its psychometric properties (52), the HDRS is one of the most widely used clinician-administered depression assessment scale and is routinely used in studies investigating ECT response (8, 53).

### Statistical and machine learning analyses

To empirically test our hypotheses, we conducted four analyses: First, we assessed the relation between empirically observed whole-brain modal ($\bar{MC})\;$and average controllability ($\bar{AC})$, respectively, and PSI during ECT. To this end, we employed an ANCOVA approach with PSI as the dependent variable, $\bar{MC}$ and $\bar{AC}$, respectively, as the independent variable. Second, to replicate the association between PSI and ECT response known from the literature, we employed an ANCOVA approach with ECT response as the dependent variable and PSI as the independent variable. Third, we tested whether pre-ECT $\bar{MC}$ and $\bar{AC}$ predicted ECT response. Again, we employed an ANCOVA approach with ECT response as the dependent variable and $\bar{MC}$ and $\bar{AC}$, respectively, as the independent variable. Note that in all of these analyses, in addition to the F-value and the one-sided *P*-value, we provide ηp^2^ as a measure of effect size. All of these analyses were conducted using the Python *statsmodels* package (statsmodels.org).

Fourth, we tested if this effect of $\bar{MC}$ and $\bar{AC}$, respectively, on ECT response is indeed mediated by PSI during ECT. To this end, we employed a mediation analysis with $\bar{MC}$ and $\bar{AC}$ as the predictor, PSI as the mediator, and ECT response as the outcome using the bias-correct, non-parametric, bootstrap-based mediation analysis implemented in the Python *pingouin* library (pingouin-stats.org). Significance of the indirect (mediation) effect was computed using the permutation test as outlined in Mac Kinnon (54) (Section 12.6) based on 10,000 permutations.

In all analyses, we included age, sex, and symptom severity before ECT as covariates. To ensure that the effects observed are not driven by basic graph properties, we additionally included the number of present edges in all analyses. In analyses involving PSI as computed from EEG during ECT, we only used data from the first ECT session as numerous studies have shown that the structural connectome as measured with DTI underlying our control analyses changes in response to previous ECT sessions (55). Note that PSI was available for 45 of the 50 patients. Thus, all analyses involving PSI are based on N = 45, whereas all other analyses are based on N = 50.

Finally, we conducted an extensive array of machine learning analyses based on structural connectome data—namely Fractional Anisotropy, Mean Diffusivity, and Number of Streamlines—to predict ECT response. Using the PHOTON AI software (56), we trained and evaluated a total of 35 machine learning pipelines. Specifically, we tested pipelines including Principal Component Analysis and univariate feature selection (with 5 and 10% thresholds) with linear and non-linear Support Vector Machines, Random Forests as well as Linear Regression. Models were trained and evaluated in a nested leave-one-out cross-validation framework and performance reported as percent variance explained by Spearman rank correlation between true and predicted treatment response. The full code can be found in the online Supplementary Material. We compared this to a simple linear regression model also using leave-one-out cross-validation based solely on the single $\bar{MC}$ and $\bar{AC}$ value, respectively, without any further optimization or model selection. Note that formally testing performance differences for significance using e.g. 1,000 permutations would require fitting 257,750,000 machine learning pipelines for the three structural connectome modalities alone and was thus not feasible given current hardware.

## Supplementary Material

pgad032_Supplementary_Data

## Data Availability

Partial restrictions to the data and/or materials apply. Anonymized data can be shared within the restrictions of the General Data Protection Regulation (GDPR) of the European Union upon request (contact: Prof. Dr. U. Dannlowski, Institute for Translational Psychiatry, University of Münster).
