# Supplementary material for: Towards a network control theory of electroconvulsive therapy response
Source: PNAS Nexus. 2023 Feb 1;2(2):pgad032. doi: 10.1093/pnasnexus/pgad032 (PMC9982063; doi:10.1093/pnasnexus/pgad032)
Supplement: pgad032_Supplementary_Data [file pgad032_supplementary_data.docx]

**Supplementary Methods:**

MRI data acquisition

Data was acquired before the first ECT session using a 3T whole body MRI scanner (Gyroscan Intera, Philips Medical Systems, Best, the Netherlands), as reported earlier [1].

T1-weighted high resolution anatomical images were acquired with a 3D fast gradient echo sequence ('Turbo Field Echo', TFE), TR=7.4 ms, TE=3.4 ms, FA=9°, 2 signal averages, inversion prepulse every 814.5ms, acquired over a field of view of 256(FH)x204(AP)x160(RL) mm, phase encoding in AP and RL direction, reconstructed to cubic voxels of 0.5x0.5x0.5 mm.

The DTI data was acquired in 36 axial slices, 3.6 mm thick with no gap (acquired matrix 128 x 128), resulting in a voxel size of 1.8 x 1.8 x 3.6 mm³. The echo time was 95 ms and the repetition time was 9473 ms. A *b*-value of 1000 sec/mm² was used for 20 diffusion-weighted images, with isotropic gradient directions plus one non-diffusion-weighted (*b* = 0 s/mm²) image. In sum, 21 images per slice were used for diffusion-tensor estimation. The total data acquisition time was approximately 8 minutes per subject. During the experiment, subjects lay supine in the MRI scanner with their head position being stabilized (Figure 1a).

Imaging Data Preprocessing

Connectomes were reconstructed using the CATO toolbox[2] following the procedure outlined in [3]. For a more detailed description of the preprocessing see [4]. In accordance with [4], we decided on using a basic DTI reconstruction rather than more advanced diffusion direction reconstruction methods to provide a reasonable balance between false negative and false positive fiber reconstructions [5]. For each subject an anatomical brain network was reconstructed, consisting of 114 cortical areas of a subdivision of the FreeSurfer’s Desikan–Killiany atlas [6, 7], and the reconstructed streamlines between these areas (Figure 1b). White matter connections were reconstructed using deterministic streamline tractography, based on the Fiber Assignment by Continuous Tracking (FACT) algorithm [8]. Network connections were included when two nodes (i.e., brain regions) were connected by at least three tractography streamlines [9]. Note that results do not substantially change if this threshold is omitted or if this threshold is increased to 10 connections. Also note that including head motion parameters from DTI did not substantially change the results. For each participant, the network information was stored in a structural connectivity matrix, with rows and columns reflecting cortical brain regions, and matrix entries representing graph edges. Edges were only described by their presence or absence to create unweighted graphs and scaled to ensure LTI model stability (for DTI quality control, see Supplementary Material).

Network Control Analysis

Building on Control Theory as the study and practice of controlling dynamical systems[10], we view the electric charge applied during ECT as the control input (u) designed to guide the system towards a seizure which characterize the output. Against this background, we apply the standard model of structural brain controllability assuming a noise-free linear time-invariant model (for a more detailed introduction, see [11, 12]):

$x\left( k+1 \right)=Ax\left( k \right)+Bu\left( k \right)$ (2)

where $x$ represents the temporal activity of 114 brain regions, $A_{114x114}$ is the adjacency matrix quantifying the structural connectivity (see *Imaging Data Preprocessing* above for details), $u(t)$ represents the input to the system (here the electric charge), and $B$ denotes the brain regions that distribute the input energy across the system (Figure 1c).

For this system, Modal Controllability ($MC$) of node $i$ is estimated as ${MC}_{i}=\sum_{j}^{114} [1-\xi_{j}^{2}(A)]v_{ij}^{2}$ where $\xi_{j}$ and $v_{ij}$ are the eigenvalues and (normalized) eigenvectors of $A$. Whole-brain modal controllability ($\bar{MC}$) is then defined as the average of nodal controllability over all nodes. Using the symmetry of $A$ this simplifies to:

$\bar{MC}= \frac{1}{114}\sum_{i}^{114} \sum_{j}^{114} \left[ 1-\xi_{j}^{2}\left( A \right) \right]v_{ij}^{2}= \frac{1}{114}\sum_{j}^{114} \left[ 1-\xi_{j}^{2}\left( A \right) \right]\sum_{i}^{114} v_{ij}^{2}=1-\frac{1}{114}\sum_{j}^{114} \xi_{j}^{2}\left( A \right)$ (3)

Note that $\bar{MC}$ is now a function of the eigenvalues only. Thus, given that the eigenvalues determine the decay rate of the response to any arbitrary input $u(t)$, equation (3) implies that whole-brain modal controllability is negatively proportional to the duration of the output signal and therefore to the output power over a fixed interval of time (note that the power of any signal $s(k)$ is defined as $P\left( u \right)= \sum_{k=0}^{N} \left\| s(k) \right\|^{2}$ where N is the length of nonzero elements of s(k)).

Also, for the system defined in (1), average controllability measures the energy content of the system’s impulse response and is defined as $Tr (W_{c})$ where $W_{c}=\sum_{t=0}^{\infty} A^{t}BB^{T}{(A^{T})}^{t}$ is the controllability Gramian of the system. Note that for a linear time-invariant system of this form, the impulse response fully determines the system’s response to any (not necessarily impulse) inputs.[13] Average controllability for a single node $i$ (i.e., B includes only one nonzero element in the $i$’th row) simplifies to:

$$AC_{i}=Tr \left( W_{c} \right)=Tr \left( B^{T}\sum_{t=0}^{\infty} A^{2t}B \right)=Tr \left( B^{T}\sum_{t=0}^{\infty} V\Xi^{2t}V^{T}B \right)=Tr \left( B^{T}V\left( I-\Xi^{2} \right)^{-1}V^{T}B \right)$$

$=\sum_{j=1}^{114} \frac{v_{\mathrm{ij}}^{2}}{1-\xi_{j}^{2}}$ (4)

where $V$ is the matrix of eigenvectors and $\Xi$ is a diagonal matrix of eigenvalues. Thus, whole-brain (i.e. mean) average controllability over all nodes becomes

$\bar{AC}= \frac{1}{114}\sum_{i=1}^{114} \sum_{j=1}^{114} \frac{v_{\mathrm{ij}}^{2}}{1-\xi_{j}^{2}}=\frac{1}{114}\sum_{j=1}^{114} \sum_{i=1}^{114} \frac{v_{\mathrm{ij}}^{2}}{1-\xi_{j}^{2}}=\frac{1}{114}\sum_{j=1}^{114} \frac{1}{1-\xi_{j}^{2}}$ (5)

Note the similarity between equation (5) and equation (3), where the effects of the eigenvectors disappear when considering the whole-brain (i.e. averaging over all regions) controllability. Also, note the opposite dependence on the system modes where larger eigenvalues increase the whole-brain average controllability and thus, opposite to the whole-brain modal controllability, is associated with longer output response and higher output power. However, we have noticed that the relation between whole-brain average and modal controllability generally follows a parabolic trajectory with extreme values for largest and smallest eigenvalues (data not shown here) and thus the relative nonlinear (nontrivial) difference between whole-brain average and modal controllability is mainly determined by the largest (slowest) and smallest (fastest) modes.

Therefore, following the hypothesis discussed in the introduction, equations (3) and (5) predict that lower whole-brain modal controllability and higher whole-brain average controllability are associated with longer output duration and thus higher signal power of the output (i.e., during the tonic-clonic seizure) and should thus result in higher PSI. Note, however, that the dominating modes in these two metrics are different (slowest dynamics for average and fastest dynamics for modal controllability). Also, the electric charge from ECT (i.e., the control input $u$) has stopped by the time PSI is calculated and only serves (in the model) to bring the brain’s state to a tonic-clonic seizure state from which the decay of the system to the postictal state can be modeled using autonomous (u=0) dynamics.

**Supplementary Material 1:**

**Study inclusion and exclusion criteria**

We recruited Caucasian subjects varying in age from 18-59 years. Patients that were treated predominantly for depressive symptoms were recruited from local psychiatric hospitals. Exclusion criteria comprised any neurological abnormalities, history of seizures, head trauma or unconsciousness, not adequately substituted hypothyroidism, severe physical impairment (e.g. cancer, instable diabetes, epilepsy etc.), pregnancy, claustrophobia, color blindness and general magnetic resonance imaging contradictions (e.g. metallic objects in the body). Further, patients with comorbid life-time diagnoses of schizophrenia, schizoaffective disorder or substance dependence were excluded.

**Calculation of medication indices**

The Medication Load Index (MedIndex [14]) was calculated as follows: We defined each psychopharmacological medication as absent (= 0), equal to no medication intake, low (= 1), meaning a dosage equal or lower than average, or high (= 2), with a dosage greater than average relative to the midpoint of the daily dose range recommended by Physician’s-Desk-Reference. We calculated the sum of all medication scores, if patients had more than one prescription. Further, chlorpromazine equivalent doses were calculated for antipsychotic medication load based on Gardner et al. [15].

**Supplementary Material 2:**

**Description of electroconvulsive therapy**

All patients started their treatment with right-sided unilateral ECT. In eight patients, treatment was converted to bilateral ECT because of insufficient clinical response to unilateral treatment. ECT monitoring included electroencephalogram (EEG), electromyogram (EMG), electrocardiogram (ECG), and blood pressure monitoring. The initial stimulus intensity was calculated using the age method. Re-stimulation, including dosage elevation in steps of 10%, was considered during single ECT sessions if the primarily induced seizure activity lasted less than 25 seconds in EEG. Due to an increasing seizure threshold throughout the course of ECT, stimulus intensity was increased in the same manner. All patients were anesthetized with methohexital sodium, propofol or sodium thiopental, and a muscle relaxant (succinylcholine) was administered.

**Table: Overview of ECT parameters.** Mean values and standard deviation of ECT stimulus and quality parameters.

| *Overview of ECT parameters* | | | |
| --- | --- | --- | --- |
| Parameters | *N* | Mean | SD |
| Number of ECT treatments | 45 | 13.0 | 4.4 |
| Average Seizure Time (EEG) | 45 | 41.9 | 10.8 |
| Average Seizure Time (EMG) | 45 | 21.1 | 8.5 |
| Postictal Suppression Index | 45 | 82.5 | 22.0 |
| Average Stimulus Charge | 45 | 55.7 | 25.4 |
| Maximum Stimulus Charge | 45 | 65.3 | 28.3 |
| Delta Stimulus Charge | 45 | 22.1 | 36.1 |
| Starting Dosage Charge | 45 | 43.22 | 15.27 |
| *Note:*  n = number of included ECT Patients, *SD* = standard deviation, EEG = electroencephalogram, EMG = electromyography | | | |

Average Seizure Time was measured by EEG in time wave-seizure activity in seconds. Average Muscle Seizure Time was measured by EMG. During ECT treatment, blood circulation of the left arm was cut off temporarily using a blood pressure cuff before Succinylcholin administration to observe muscle seizure activity. Postictal Suppression Index measures successful inhibition of seizure activity calculated by the ratio of EEG amplitude before and after the seizure ceases. Maximum Stimulus Charge is reported as percentage value of 504 mC (millicoulomb). ΔStimulus Charge is calculated by subtracting the applied charge during the first ECT from the applied charge during last ECT and is therefore a measure of how much applied charge had to be adjusted, an indicator of poor seizure quality throughout the treatment. Indeces were calculated automatically by the ECT instrument (Thymatron system IV; Somatics Inc).

**Supplementary Material 3: Medication and comorbid disorder details in patient group.**

| Characteristics | ECT^1^ (*n* = 50) |
| --- | --- |
| T_0 (before ECT treatment)_ |  |
| Medication load | 3.26 ± 2.08 |
| Antidepressants |  |
| NaSSA | 8 |
| Tricyclics | 5 |
| NDRI | 1 |
| SSRI | 3 |
| SSNRI | 19 |
| MAO-Inhibitors | 1 |
| Other | 6 |
| Antipsychotics | 23 |
| Mood stabilizer | 7 |
| T_1 (after ECT treatment)_ |  |
| Medication load | 3.58 ± 2.29 |
| Antidepressants |  |
| NaSSA | 10 |
| Tricyclics | 3 |
| NDRI | 2 |
| SSRI | 2 |
| SSNRI | 21 |
| MAO-Inhibitors | 0 |
| Other | 4 |
| Antipsychotics | 22 |
| Mood stabilizer | 5 |
| T_0_-T_1_ |  |
| ΔMedication load | -0.32 ± 1.63 |
| (repeated measures t-test not significant: t(df=49)= 1.36, p= .17) | |
| Depression subtype |  |
| Psychotic depression | 2 |
| Co-morbid disorders |  |
| GAD | 1 |
| Panic/Agoraphobia | 17 |
| Specific phobia | 2 |
| PTSD | 4 |
| OCD | 1 |
| Social phobia | 7 |
| Dysthymia | 3 |
| Eating disorder | 5 |
| Substance abuse | 12 |

**Supplementary Material 4: DTI quality control**

Measures for outlier detection included 1. average number of streamlines, 2. average fractional anisotropy, 3. average prevalence of each subject’s connections (low value, if the subject has “odd” connections), and 4. average prevalence of each subjects connected brain regions (high value, if the subject misses commonly found connections). For each metric the quartiles (Q1, Q2, Q3) and the interquartile range (IQR=Q3-Q1) was computed across the group and a datapoint was declared as an outlier if its value was below Q1-1.5*IQR or above Q3+1.5*IQR on any of the four metrics. This led to the exclusion of three subjects. Note that results do not substantially change of these three outliers are not removed.

**Supplementary Material 5:**

Code running the PHOTON AI Machine Learning approach applied separately for Fractional Anisotropy, Mean Diffusivity, and Number of Streamlines derived from each patients structural connectome.

from photonai.base import Hyperpipe, PipelineElement, OutputSettings, Switch
from sklearn.model_selection import LeaveOneOut

pipe = Hyperpipe('photon_ANALYSIS_NAME', optimizer='grid_search',
 metrics=['mean_squared_error'], best_config_metric='mean_absolute_error',
 inner_cv=LeaveOneOut(), outer_cv=LeaveOneOut(),
 calculate_metrics_across_folds=True, calculate_metrics_per_fold=False,
 random_seed=True, verbosity=1,
 project_folder='./tmp/', output_settings=OutputSettings(overwrite_results=True))

 """ add imputer and scaler """
 pipe += PipelineElement('VarianceThreshold')
 pipe += PipelineElement('SimpleImputer')
 pipe += PipelineElement('RobustScaler')

 """ add transformer elements """
 transformer_switch = Switch('TransformerSwitch')
 transformer_switch += PipelineElement('PCA', hyperparameters={'n_components': None}, test_disabled=True)
 transformer_switch += PipelineElement('FRegressionSelectPercentile', hyperparameters={'percentile': [5, 10]}, test_disabled=True)
 pipe += transformer_switch

 """ add estimator elements """
 estimator_switch = Switch('EstimatorSwitch')
 estimator_switch += PipelineElement('LinearSVR', hyperparameters={'C': [1e-8, 1e-4, 1, 4]})
 estimator_switch += PipelineElement('SVR', kernel='rbf')
 estimator_switch += PipelineElement('RandomForestRegressor')
 estimator_switch += PipelineElement('LinearRegression')

 pipe += estimator_switch
 results = pipe.fit(X, targets)

**Supplementary Material 6: Results**

In addition to the Leave-One-Out Cross Validation (CV), we also conducted a 5-fold CV. As is to be expected when bias increases due to lower training set size, average performance estimates decreased for all measures. Importantly, as this effect occurs for the controllability-based as well as the ML-based prediction, controllability metrics still perform comparable to ML models as before. Specifically, we showed that $\bar{MC}$ (r^2^=5.33%) as well as $\bar{AC}$ (r^2^=3.55%), respectively, explain nominally more variance in treatment response than the best machine learning model for each modality (Fractional Anisotropy r^2^=1.28%; Mean Diffusivity r^2^=2.82%; Number of Streamlines r^2^<0.01%).

**Supplementary References**

1. Repple J, Meinert S, Grotegerd D, Kugel H, Redlich R, Dohm K, et al. A voxel-based diffusion tensor imaging study in unipolar and bipolar depression. Bipolar Disord. 2017;19:23–31.

2. Lange SC de, Heuvel MP van den. Structural and functional connectivity reconstruction with CATO - A Connectivity Analysis TOolbox. BioRxiv. 2021:2021.05.31.446012.

3. Collin G, van den Heuvel MP, Abramovic L, Vreeker A, de Reus MA, van Haren NEM, et al. Brain network analysis reveals affected connectome structure in bipolar I disorder. Hum Brain Mapp. 2016. 2016. https://doi.org/10.1002/hbm.23017.

4. Repple J, Mauritz M, Meinert S, de Lange SC, Grotegerd D, Opel N, et al. Severity of current depression and remission status are associated with structural connectome alterations in major depressive disorder. Mol Psychiatry. 2020;25:1550–1558.

5. Sarwar T, Ramamohanarao K, Zalesky A. Mapping connectomes with diffusion MRI: deterministic or probabilistic tractography? Magn Reson Med. 2019;81:1368–1384.

6. Hagmann P, Cammoun L, Gigandet X, Meuli R, Honey CJ, Van Wedeen J, et al. Mapping the structural core of human cerebral cortex. PLoS Biol. 2008. 2008. https://doi.org/10.1371/journal.pbio.0060159.

7. Cammoun L, Gigandet X, Meskaldji D, Thiran JP, Sporns O, Do KQ, et al. Mapping the human connectome at multiple scales with diffusion spectrum MRI. J Neurosci Methods. 2012. 2012. https://doi.org/10.1016/j.jneumeth.2011.09.031.

8. Mori S, Van Zijl PCM. Fiber tracking: Principles and strategies - A technical review. NMR Biomed. 2002.

9. de Reus MA, van den Heuvel MP. Estimating false positives and negatives in brain networks. Neuroimage. 2013;70:402–409.

10. Thomas PJ, Olufsen M, Sepulchre R, Iglesias PA, Ijspeert A, Srinivasan M. Control theory in biology and medicine: Introduction to the special issue. Biol Cybern. 2019;113:1–6.

11. Gu S, Pasqualetti F, Cieslak M, Telesford QK, Yu AB, Kahn AE, et al. Controllability of structural brain networks. Nat Commun. 2015;6:1–10.

12. Tang E, Bassett DS. Colloquium: Control of dynamics in brain networks. Rev Mod Phys. 2018;90:31003.

13. Oppenheim A V, Willsky AS, Nawab SH. Signals and systems Prentice Hall. Inc, Up Saddle River, New Jersey. 1997;7458.

14. Redlich R, Almeida JRC, Grotegerd D, Opel N, Kugel H, Heindel W, et al. Brain morphometric biomarkers distinguishing unipolar and bipolar depression: a voxel-based morphometry-pattern classification approach. JAMA Psychiatry. 2014;71:1222–1230.

15. Gardner DM, Murphy AL, O’Donnell H, Centorrino F, Baldessarini RJ. International consensus study of antipsychotic dosing. Am J Psychiatry. 2010;167:686–693.
